# Ultra-Low Temperature Cryopreservation of Sperm from a New Type of Hybrid Bream

**DOI:** 10.3390/ijms27177726

**Published:** 2026-08-28

**Authors:** Wei Zeng, Xinxing Zheng, Yating Zhu, Jiao Wang, Minglin Dong, Can Yang, Yuqin Shu, Conghui Yang, Yi Zhou

**Affiliations:** 1 Engineering Research Center of Polyploid Fish Reproduction and Breeding of the State Education Ministry, College of Life Sciences, Hunan Normal University, Changsha 410081, China; zengwei_2014@163.com (W.Z.); xinxing_zheng@163.com (X.Z.); zhuyating202303@163.com (Y.Z.); 15338044347@163.com (J.W.); minglin2000@foxmail.com (M.D.); 17773712681@163.com (C.Y.); shuyuqin@hunnu.edu.cn (Y.S.); 2 Yuelushan Laboratory, Changsha 410082, China

**Keywords:** cryopreservation, Hefang bream, sperm quality, proteomics, ultrastructure

## Abstract

Sperm cryopreservation is crucial for artificial propagation and the long-term preservation of valuable germplasm resources. This study aimed to establish an effective ultra-low temperature sperm cryopreservation protocol for Hefang bream (HFB), a novel hybrid bream variety developed through distant hybridization between blunt snout bream (*Megalobrama amblycephala*) and topmouth culter (*Culter alburnus*) followed by two rounds of backcrossing. Different combinations of extenders and cryoprotectants were evaluated based on post-thaw sperm motility and motion parameters to identify the optimal cryoprotective formulation for HFB sperm, with untreated fresh sperm as the control. Fresh sperm exhibited a total motility (MOT) of 98.87 ± 1.40%, curvilinear velocity (VCL) of 118.87 ± 5.38 μm/s, straight-line velocity (VSL) of 86.18 ± 5.67 μm/s, and average path velocity (VAP) of 107.48 ± 4.23 μm/s. The optimal formulation consisted of D15 extender (composed of 8 g/L NaCl, 0.5 g/L KCl and 15 g/L glucose) supplemented with 10% dimethyl sulfoxide (DMSO). Using a fresh sperm-to-cryoprotective medium ratio of 1:5 and a stepwise cooling procedure, post-thaw MOT, VCL, VSL, and VAP were 43.77 ± 13.85%, 37.54 ± 3.06 μm/s, 29.82 ± 1.98 μm/s, and 31.99 ± 2.14 μm/s, respectively. Further analyses revealed that the plasma membrane integrity (PMI), DNA integrity (DI) and mitochondrial activity (MA) of cryopreserved sperm were 43.40 ± 2.01%, 57.10 ± 4.79%, and 42.00 ± 1.65%, respectively, all of which were significantly lower than those of fresh sperm (*p* < 0.05). Ultrastructural analyses using scanning electron microscopy (SEM) and transmission electron microscopy (TEM) demonstrated that cryopreserved spermatozoa exhibited structural abnormalities, including plasma membrane disruption, flagellar fragmentation, and mitochondrial damage. Artificial insemination experiments showed that the fertilization and hatching rates of the cryopreserved sperm group were 55.10 ± 3.30% and 81.50 ± 3.29%, respectively, indicating that the established protocol could effectively support artificial reproduction in HFB. Furthermore, proteomic analysis was performed to investigate cryopreservation-induced molecular damage, identifying 7 cryopreservation-associated leakage proteins that were mainly enriched in pathways related to energy metabolism, cytoskeletal organization, and oxidative stress response. This study establishes an effective sperm cryopreservation protocol for HFB and provides insights into the cellular and molecular mechanisms underlying cryopreservation-induced sperm damage, offering a valuable reference for the development of sperm cryopreservation technologies in other economically important fish species.

## 1. Introduction

The Hefang bream (HFB) is a novel hybrid bream variety developed through distant hybridization, specifically through two rounds of backcrossing following the initial cross between blunt snout bream (*Megalobrama amblycephala*) and topmouth culter (*Culter alburnus*) [1,2]. Morphologically, HFB resembles blunt snout bream, but it exhibits several superior production traits, including a herbivorous feeding habit, rapid growth, strong tolerance to hypoxia, and tender flesh [3,4]. Notably, HFB overcomes the reproductive barriers commonly associated with distant hybridization, as both male and female individuals exhibit complete fertility, representing a valuable “bisexually fertile” breeding line [1]. These advantageous characteristics make HFB not only a superior aquaculture variety but also a valuable germplasm resource with considerable breeding potential, owing to its unique genetic background derived from distant hybridization. Particularly, male HFB individuals possess well-developed testes and undergo complete spermatogenesis, producing milky and viscous semen that can be readily collected by stripping and exhibits normal fertilization capacity [5]. These reproductive characteristics provide a strong biological foundation for the long-term preservation and utilization of the HFB variety. Therefore, establishing an effective technological system for HFB germplasm conservation and utilization is of great importance for maintaining its superior genetic characteristics and enhancing aquaculture performance. Among various germplasm conservation approaches, sperm cryopreservation represents a practical and efficient strategy enabling the long-term storage and efficient utilization of valuable aquatic resources.

Ultra-low temperature cryopreservation effectively suppresses cellular biochemical and metabolic activities in sperm, allowing the restoration of sperm viability and fertilization capacity after thawing and thereby enabling stable long-term storage of male germplasm resources [6]. This technology can overcome temporal and spatial constraints associated with reproductive cycles and geographic separation, providing essential support for fish breeding programs. Since Blaxter successfully preserved Atlantic herring (*Clupea harengus*) sperm at low temperature (−79 °C) in 1953, substantial advances have been achieved in fish sperm cryopreservation [7]. This technique has not only provided an effective approach for the preservation of valuable germplasm resources in numerous economically important fish species, but has also contributed significantly to the conservation of rare and endangered species through the establishment of sperm banks [8,9,10,11]. However, the successful application of sperm cryopreservation remains highly species-specific and requires careful optimization of cryoprotective agents and freezing protocols based on post-thaw sperm quality evaluation.

The key challenge in sperm cryopreservation is to minimize ice crystal-induced damage during the critical temperature ranges (−130 to −196 °C and 0 to −60 °C), within which intracellular free water can form needle-like ice crystals, leading to mechanical disruption of cell membranes and organelles, particularly mitochondria. The rapid cooling conditions provided by liquid nitrogen promote water vitrification and reduce crystallization-related injury [8]. Furthermore, the addition of cryoprotective agents can effectively reduce the extent of these critical temperature ranges and alleviate cryopreservation-induced damage [12]. Sperm cryoprotective media generally consist of extenders and cryoprotectants. Permeating cryoprotectants are typically small-molecule compounds that can penetrate the sperm plasma membrane and enter spermatozoa, where they exert protective effects by reducing the freezing point, inhibiting intracellular ice formation, and stabilizing cellular proteins; meanwhile, their low cytotoxicity and high water solubility are essential for effective cryoprotection [13]. Extenders are used to maintain an isotonic or slightly hypertonic environment relative to seminal plasma, preventing premature sperm activation before cryopreservation, while providing a suitable buffering environment for the incorporation of cryoprotectants [14]. Commonly used cryoprotectants include organic solvents such as DMSO, methanol (MeOH), glycerol (GLY), and ethylene glycol (EG), whereas extenders typically contain salts (e.g., NaCl), sugars (e.g., glucose and trehalose), lipids (e.g., phospholipids), proteins, glycine, and antibiotics [15,16]. Due to interspecific variations in sperm cellular structure and physiological characteristics, the optimal composition and concentration of cryoprotective media differ among fish species and therefore require species-specific optimization [14,17].

Previous studies have been conducted on sperm cryopreservation in culter–bream fishes. In blunt snout bream, frozen sperm samples preserved using D15 extender supplemented with 8–12% DMSO showed stable post-thaw sperm motility ranging from 55% to 65% [18]. For triangular bream (*Megalobrama terminalis*), a cryoprotective solution containing 20% DMSO in Hank’s solution was effective for sperm cryopreservation, achieving a sperm activation rate of 60.33% [19]. Similarly, in topmouth culter, D15 extender combined with 10% EG resulted in a post-thaw sperm motility of 62.67% [20]. In addition, ultra-low temperature sperm cryopreservation has also been investigated in hybrid fish, a special group among aquaculture species. For example, the optimal cryoprotective medium for the F_1_ hybrid derived from female topmouth culter × male triangular bream consisted of 15% DMSO supplemented with Hank’s solution, which differed from those used for both parental species [19]. In contrast, the optimal cryoprotective formulation for the hybrid fish derived from female white crucian carp (*Carassius cuvieri*) × male red crucian carp (*C. auratus* red var.) was identical to that of the maternal species, consisting of D14 extender supplemented with 15% DMSO, whereas the paternal species required D20 extender with 10% DMSO [21]. These findings suggest that sperm cryopreservation requirements in hybrid fish may differ substantially from those of their parental species, and that parental cryopreservation protocols should be regarded as references rather than directly applicable protocols.

Accurate evaluation of post-thaw sperm quality is essential for assessing the effectiveness of sperm cryopreservation and optimizing cryopreservation protocols. Currently, computer-assisted sperm analysis (CASA) systems are widely employed to objectively evaluate post-thaw sperm motility by measuring various motion parameters, including MOT and VAP [22]. During cryopreservation, sperm movement velocity is often severely impaired, but this reduction can be alleviated through optimization of cryopreservation conditions [23]. In addition to motility assessment, sperm structural integrity is closely associated with fertilization capacity. Microscopic imaging techniques, including SEM and TEM, have revealed that cryopreserved spermatozoa may undergo structural damage, such as flagellar detachment, disruption of the midpiece sheath, mitochondrial disorganization, and shrinkage or deformation of the head plasma membrane [24,25]. Furthermore, the integrity of key cellular components, including the plasma membrane, mitochondria, and DNA, can be evaluated to further characterize post-thaw sperm quality [26]. Fluorescence staining combined with flow cytometry provides a high-throughput approach for quantitative assessment of these cellular characteristics. To further elucidate the mechanisms underlying cryopreservation-induced damage, enzymatic activity assays, transcriptomic sequencing, and proteomic analyses have been applied to investigate alterations in sperm metabolic and antioxidant defense systems. Previous studies have demonstrated that cryopreservation-induced mechanical damage and membrane disruption may cause the leakage of intracellular enzymes, resulting in reduced enzyme activity within spermatozoa but increased enzyme activity in seminal plasma [27]. Ultimately, cryopreservation may compromise fertilization capacity by impairing sperm structure and functional activity. Therefore, fertilization assays remain an indispensable approach for post-thaw sperm evaluation, as they directly determine the ability of cryopreserved sperm to fertilize oocytes and support normal embryo development.

The present study aimed to establish an effective ultra-low temperature cryopreservation protocol for HFB sperm. Different combinations of extenders and cryoprotectants were evaluated based on post-thaw sperm motility and motion parameters. Post-thaw sperm quality was comprehensively evaluated through analyses of sperm morphology, ultrastructure, plasma membrane integrity (PMI), DNA integrity (DI), mitochondrial activity (MA), fertilization performance, and hatching success. Additionally, proteomic analysis was performed to investigate changes in sperm protein expression profiles and identify biological pathways associated with cryopreservation-induced damage. The findings provide a theoretical and technical foundation for the long-term conservation and artificial propagation of HFB germplasm resources and contribute to the development of sperm cryopreservation strategies for other economically important fish species.

## 2. Results

### 2.1. Effects of Different Extender on Sperm Motility

Sperm density of HFB was determined by hemocytometer counting, with a value of (15.80 ± 1.50) × 10^9^ cells/mL. On this basis, a CASA system was applied to quantify sperm motility and kinematic characteristics. The motility of fresh semen reached 98.87 ± 1.40%. At five minutes post-dilution, sperm motility in the Kurokura group was 77.8 ± 7.60%, which was significantly lower than that of fresh sperm. By contrast, no significant differences in motility were detected between the D1, D15, and D20 extender groups and the fresh semen. After 24 h of storage, all sperm in the fresh control and Kurokura groups were completely immotile. Among the three extenders, the D15 group exhibited the highest motility of 98.37 ± 1.48%, followed by the D1 group at 97.1 ± 2.15%. The motility of the D20 group decreased significantly to 91.13 ± 3.17%. At 120 h post-storage, motile sperm were only observed in the D15 group, with a motility percentage of 14.92 ± 2.25% (Table 1). In addition, the VCL, VSL, and VAP of fresh sperm were 118.87 ± 5.38 μm/s, 86.18 ± 5.67 μm/s, and 107.48 ± 4.23 μm/s, respectively. At five minutes post-dilution, the three sperm kinematic parameters in the D1 and D15 groups did not differ significantly from those in the fresh control. In contrast, the D20 group showed markedly lower VSL than fresh sperm, while those in the Kurokura group were significantly lower than those in fresh semen. After 24 h, the sperm in both the fresh semen group and the Kurokawa group had completely died. At 120 h of storage, only sperm preserved in D15 extender possessed detectable kinematic parameters, including VCL of 41.06 ± 4.45 μm/s, VSL of 28.61 ± 2.92 μm/s, and VAP of 38.84 ± 4.69 μm/s (Table 2). The above data verified that D15 was the optimal extender for HFB semen preservation.

### 2.2. Effects of CPAs on Cryopreservation Efficiency

**Figure 1:**
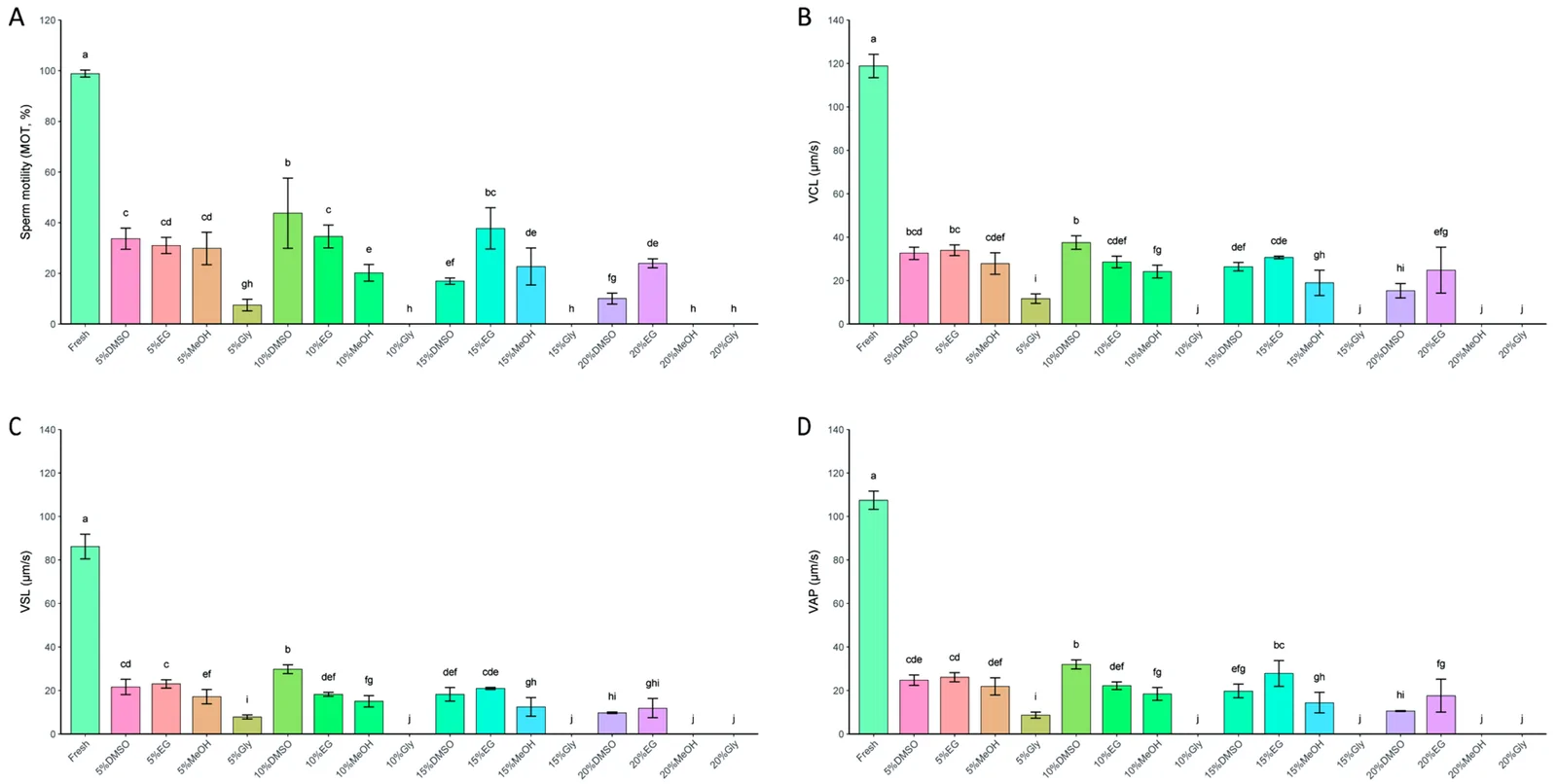
Effects of different types and concentrations of cryoprotectants on sperm motility parameters. (**A**) MOT; (**B**) VCL; (**C**) VSL; (**D**) VAP. Different lowercase letters indicate significant differences among groups (*p* < 0.05).

D15 was used as the base diluent for sperm cryopreservation, in combination with four permeating cryoprotectants, each tested across a series of concentrations (5%, 10%, 15%, 20%). Post-thaw sperm motility was highest in the 10% DMSO group (43.77 ± 13.85%), followed by the 15% EG group (37.77 ± 8.11%). No viable sperm were recovered after thawing in the 10%, 15%, and 20% Gly groups, nor in the 20% MeOH group (Figure 1A). All three kinematic parameters (VCL, VSL, and VAP) decreased significantly following freeze-thawing across all viable treatment groups. The 10% DMSO group maintained the highest post-thaw kinematic performance, with VCL, VSL, and VAP reaching 37.54 ± 3.06, 29.82 ± 1.98, and 31.99 ± 2.14 μm/s, respectively. This was followed by the 5% EG group (VCL: 33.98 ± 2.48 μm/s; VSL: 23.00 ± 1.93 μm/s) and the 15% EG group (VAP: 27.87 ± 5.94 μm/s). No velocity parameters were detectable in the 10%, 15%, and 20% Gly groups or the 20% MeOH group post-thaw (Figure 1B–D). Results indicated that 10% DMSO in the diluent provided the best cryoprotective effect.

### 2.3. Effects of Cryopreservation with D15 Diluent Supplemented with 10% DMSO on Sperm Quality

**Figure 2:**
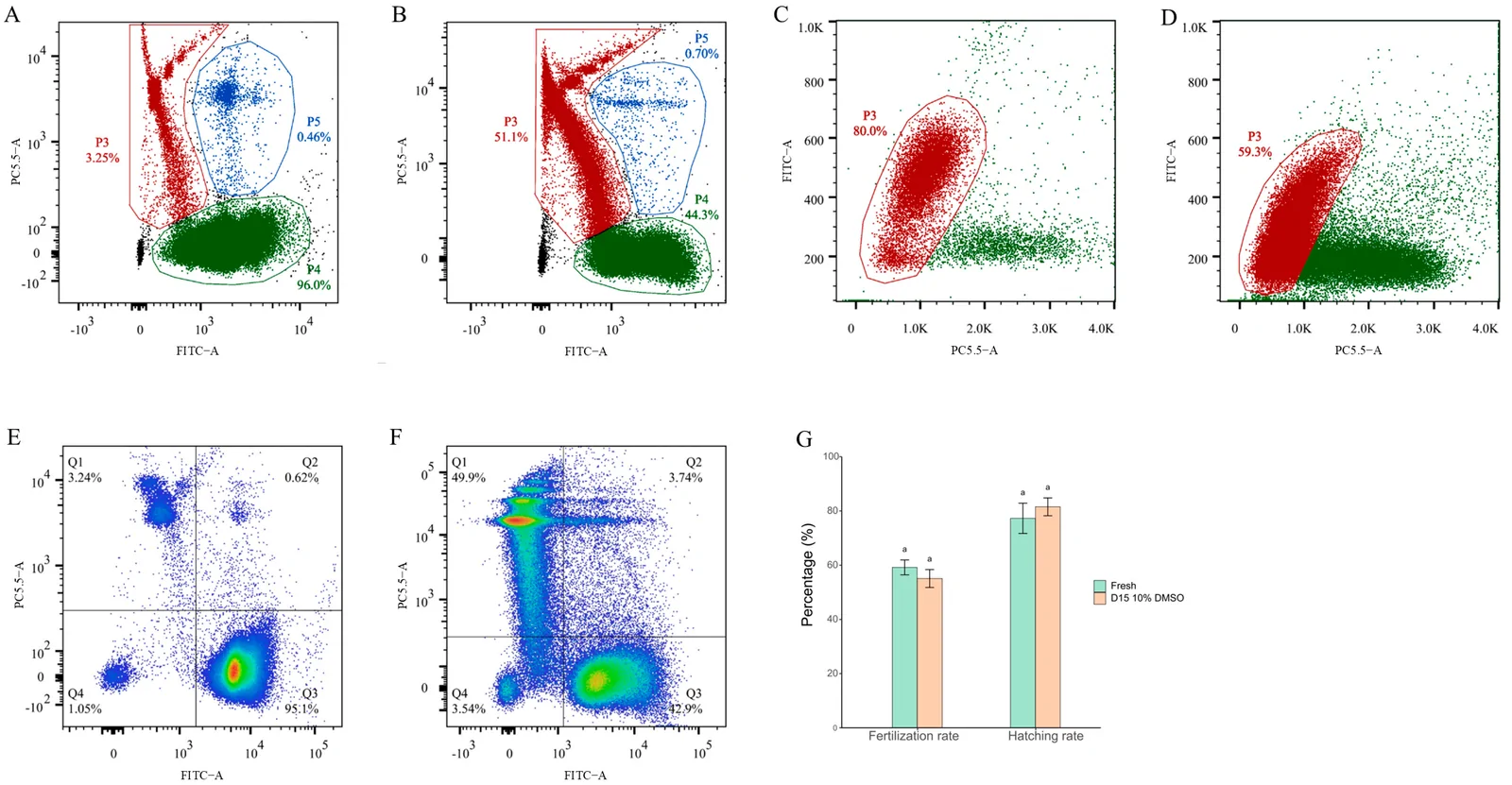
Flow cytometric evaluation of sperm quality and assessment of fertilization capacity in HFB sperm. (**A**,**B**) Dot plots of SYBR−14/PI-stained sperm for PMI evaluation. The FITC−A channel records SYBR-14 green fluorescence signal corresponding to live cell nucleic acid staining; the PC5.5−A channel records PI red fluorescence signal corresponding to membrane-compromised dead sperm. (**A**) PMI of fresh sperm detected by flow cytometry; (**B**) PMI of frozen-thawed sperm detected by flow cytometry. P3: Sperm with damaged plasma membrane and dead (SYBR−14^−^/PI^+^); P4: Sperm with intact plasma membrane (SYBR−14^+^/PI^−^); P5: Sperm in a transitional state of impending death (SYBR−14^+^/PI^+^). (**C**,**D**) Dot plot diagrams of AO-stained sperm for DI evaluation. The FITC−A channel records AO green fluorescence signal corresponding to double-stranded DNA; the PC5.5−A channel records AO red/orange fluorescence signal corresponding to single-stranded or fragmented DNA. (**C**) DI of fresh sperm detected by flow cytometry; (**D**) DI of frozen-thawed sperm detected by flow cytometry. P3: Sperm with intact DNA. (**E**,**F**) Density plots of Rh123/PI-stained sperm for MA evaluation. The FITC-A channel reports Rh123 green fluorescence signal for high mitochondrial activity; the PC5.5−A channel reports PI red fluorescence signal for membrane-compromised dead sperm. (**E**) MA of fresh sperm detected by flow cytometry; (**F**) MA of frozen-thawed sperm detected by flow cytometry. Q1: Sperm with inactive mitochondria (Rh123^−^/PI^+^); Q2: Sperm in a transitional state of impending death but with active mitochondria (Rh123^+^/PI^+^); Q3: Live sperm with normal mitochondrial function (Rh123^+^/PI^−^); Q4: Sperm without mitochondrial transmembrane potential (Rh123^−^/PI^−^). (**G**) Fertilization rate and hatching rate of HFB sperm. Values same lowercase letter indicate no significant difference.

Flow cytometry analysis with SYBR-14/propidium iodide (SYBR-14/PI) double staining revealed that the PMI of fresh sperm was 96.63 ± 1.29%, whereas the PMI of post-thaw sperm cryopreserved with D15 + 10% DMSO was 43.40 ± 2.01%, representing a significant decrease compared with fresh sperm (Figure 2A,B). Assessment of DI via acridine orange (AO) staining by flow cytometry showed that fresh sperm had a DI value of 79.33 ± 2.27%, while post-thaw sperm exhibited a significantly lower DI of 57.10 ± 4.79% (Figure 2C,D). As determined by rhodamine 123/PI (Rh123/PI) double staining, the MA of fresh sperm was 95.27 ± 1.16%, which was significantly higher than that of post-thaw sperm (42.00 ± 1.65%; Figure 2E,F). Despite the significant declines in all cellular-level quality parameters detected by flow cytometry, the reproductive function of post-thaw sperm remained well preserved. The fertilization rate and hatching rate of fresh sperm were 59.20 ± 2.75% and 77.27 ± 5.58%, respectively. For post-thaw sperm cryopreserved with D15 + 10% DMSO, the fertilization rate and hatching rate were 55.10 ± 3.30% and 81.50 ± 3.29%, respectively. Neither parameter differed statistically from those of fresh sperm (Figure 2G).

### 2.4. Effects of Cryopreservation on Sperm Ultrastructure

**Figure 3:**
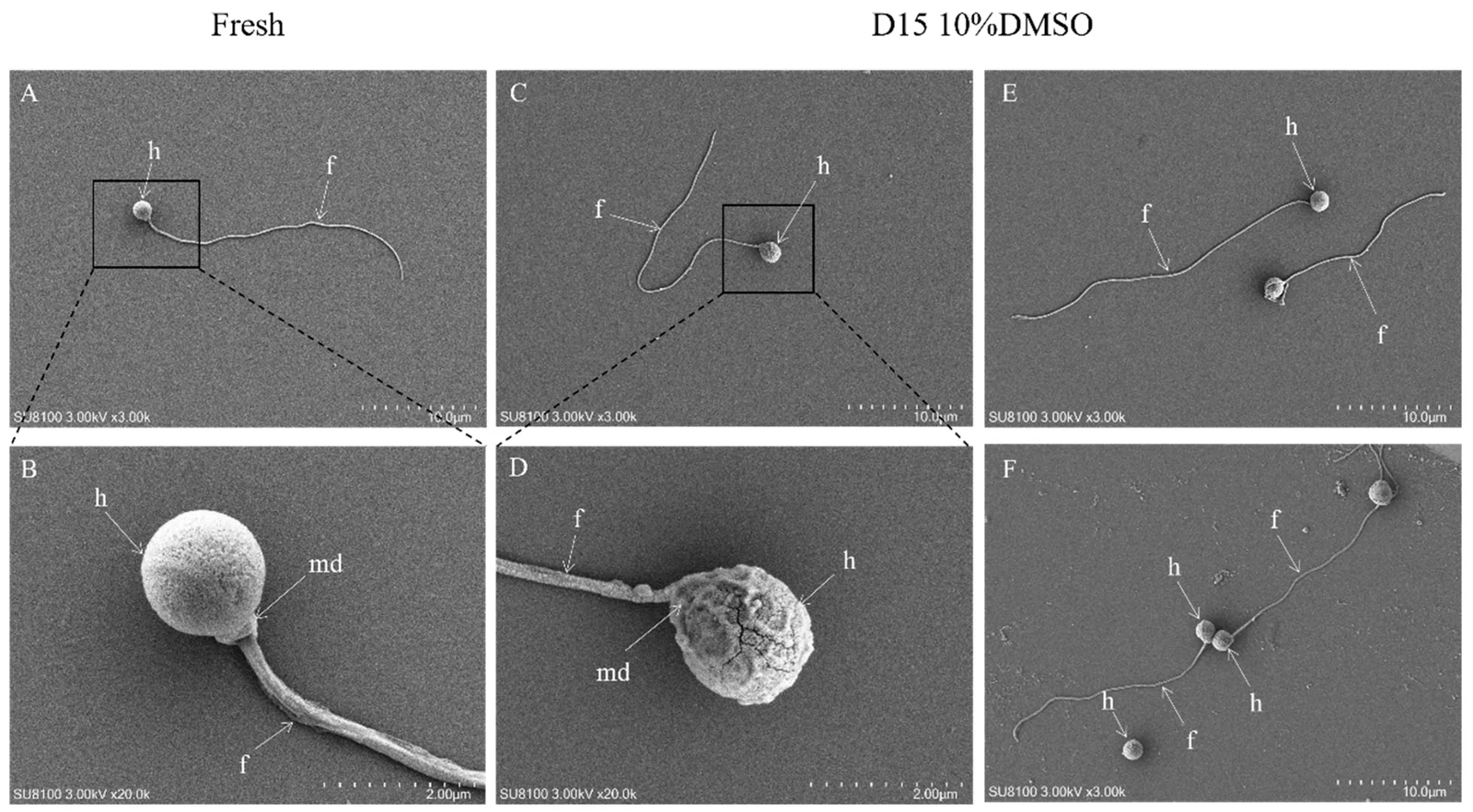
Observation on the ultrastructure of HFB sperm before and after ultra-low temperature freezing by SEM. (**A**) Panoramic view of fresh sperm at low magnification. (**B**) High-magnification view of the boxed region in (**A**). (**C**) Panoramic view of post-thaw sperm from the D15 + 10% DMSO cryopreservation group at low magnification. (**D**) High-magnification view of the boxed region in (**C**). (**E**,**F**) Panoramic views of post-thaw sperm from the D15 + 10% DMSO cryopreservation group at low magnification. h: sperm head; md: midpiece of sperm; f: flagellum.

**Figure 4:**
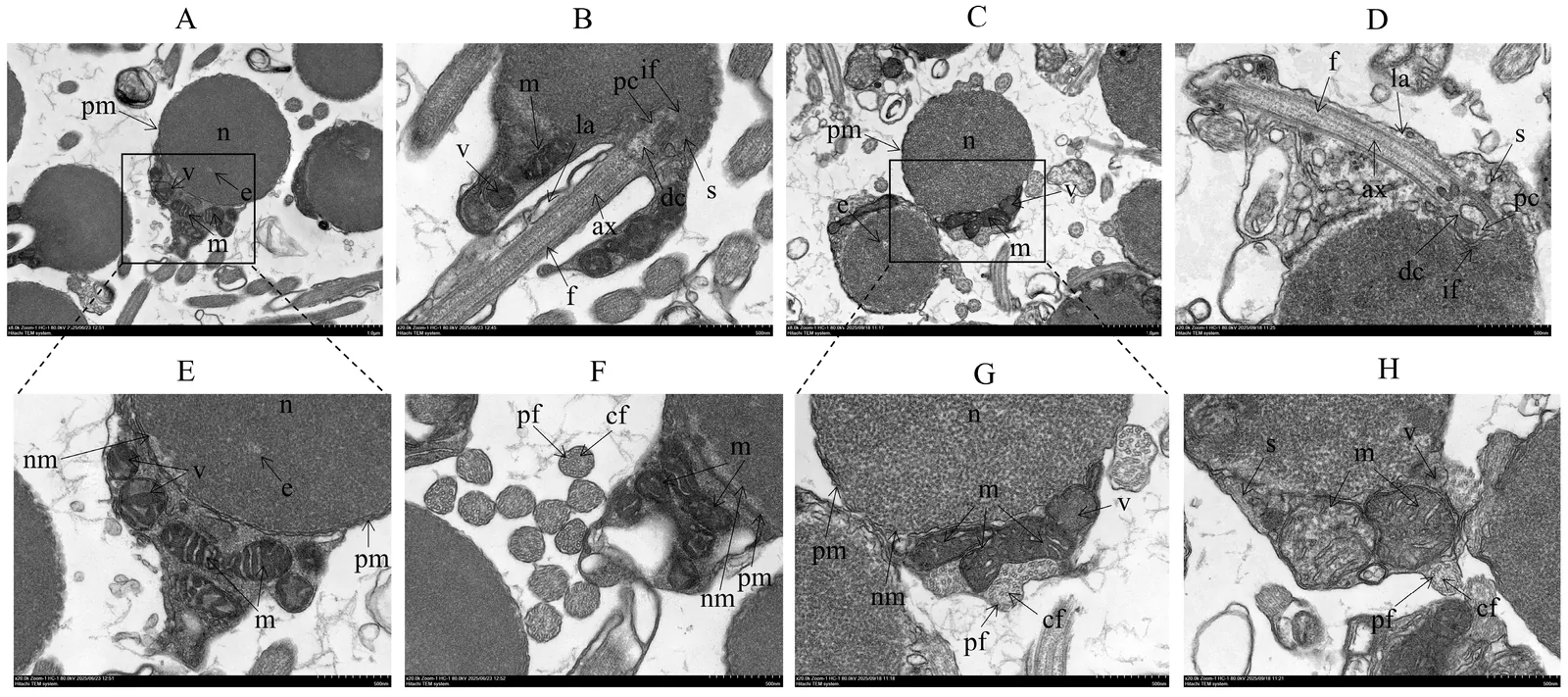
Observation of the ultrastructure of HFB sperm before and after ultra-low temperature freezing by TEM. (**A**) Low-magnification view of the head, midpiece and flagellum of fresh sperm in longitudinal section. (**B**) High-magnification view of the midpiece and flagellum of fresh sperm in longitudinal section. (**C**) Low-magnification view of the head, midpiece and flagellum of post-thaw sperm from the D15 + 10% DMSO cryopreservation group in longitudinal section. (**D**) High-magnification view of the midpiece and flagellum of post-thaw sperm from the D15 + 10% DMSO cryopreservation group in longitudinal section. (**E**) High-magnification view of the boxed region in (**A**). (**F**) High-magnification view of the longitudinal section of the midpiece and the transverse sections of the flagella of fresh sperm. (**G**) High-magnification view of the boxed region in (**C**). (**H**) High-magnification view of the longitudinal section of the midpiece and the transverse sections of the flagella of post-thaw sperm from the D15 + 10% DMSO cryopreservation group. n: nucleus; pm: plasma membrane; nm: nuclear membrane; e: electron-lucent region; m: mitochondrion; cf: central fiber; pf: peripheral fiber; s: sleeve cavity; if: implantation fossa; f: flagellum; v: vesicle; la: lateral fin; ax: axoneme; pc: proximal centriole; dc: distal centriole.

Sperm ultrastructure before and after cryopreservation was characterized using SEM and TEM. Fresh sperm displayed intact morphology typical of teleost fish. SEM showed that the sperm consisted of three parts: the head (h), the midpiece (md), and the tail, with a total length of about 30.1 μm. The acrosome-less spherical head (≈1.7 μm in diameter) had a smooth surface and an intact plasma membrane (pm). The midpiece was tightly connected to the head, and the slender flagellum (f) (≈28.4 μm in length, 0.3 μm in diameter) was fully extended with a continuous membrane structure (Figure 3A,B). Under TEM, the internal structure was well-organized. The nucleus occupied most of the head volume, with dense, uniformly electron-dense chromatin. The plasma membrane and nuclear membrane remained intact and continuous. The midpiece contained a centriolar complex and sleeve structure, with regularly oval mitochondria and moderate vesicles distributed around the axoneme. The flagellum exhibited a canonical “9 + 2” axonemal arrangement, with clearly layered fibrous sheaths, peripheral fibers (pf), central fibers (cf), and tightly associated lateral fins (Figure 4A,B,E,F).

After cryopreservation, varying degrees of ultrastructural damage were observed. The SEM results showed that the sperm heads exhibited contraction, implantation fossa (if), and even cracks, with irregularly folded contours and some of the plasma membranes detaching. The midpiece exhibited membrane damage and swelling, and the head–midpiece junction was disrupted in some sperm. The centriolar complex was located in the implantation fossa, including the proximal centriole (pc) and the distal centriole (dc). The flagellum became curled, folded, or fractured, with a rough and discontinuous membrane (Figure 3C–F). The results of TEM indicated that the freezing damage included deformation and rupture of the plasma membrane, loss of cytoplasm, local contraction of the nuclear membrane, and uneven electron density of chromatin accompanied by obvious reticular interstices. Organelle aggregation, an increase in vesicles, and swollen mitochondria with broken, blurred, or absent cristae were also evident. Moreover, the flagellum was frequently separated from the head and midpiece, with disorganized peripheral and central fibers, loose and detached lateral fins, and impaired integrity of motility-related substructures (Figure 4C,D,G,H).

### 2.5. 4D-DIA Quantitative Proteomics Identifies Cryostress-Induced Sperm Leakage Proteins

**Figure 5:**
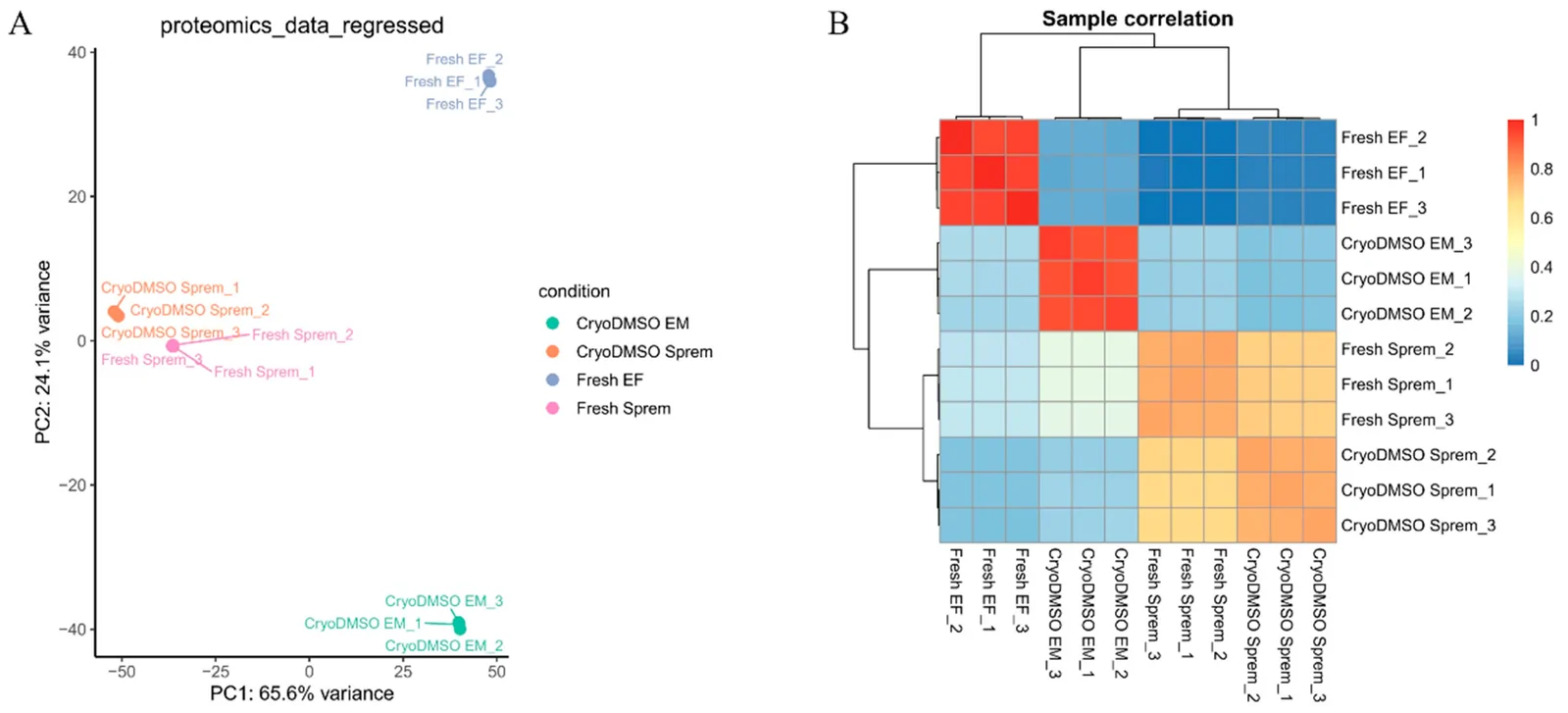
Analysis of protein differences and correlation between fresh sperm and frozen-thawed sperm samples. (**A**) PCA of proteins in fresh sperm and thawed sperm samples; (**B**) Hierarchical clustering analysis of proteins in fresh sperm and thawed sperm samples.

A systematic analysis was performed on fresh EF, CryoDMSO EM, fresh sperm, and CryoDMSO Sperm using 4D-DIA quantitative proteomic technology. Principal component analysis (PCA) showed that the samples exhibited clear grouping and clustering on the first principal component (PC1, 65.6% variation) and the second principal component (PC2, 24.1% variation) (Figure 5A). The sample correlation heatmap revealed a high degree of consistency among biological replicates within each group, alongside marked transcriptional divergence between groups (Figure 5B).

**Figure 6:**
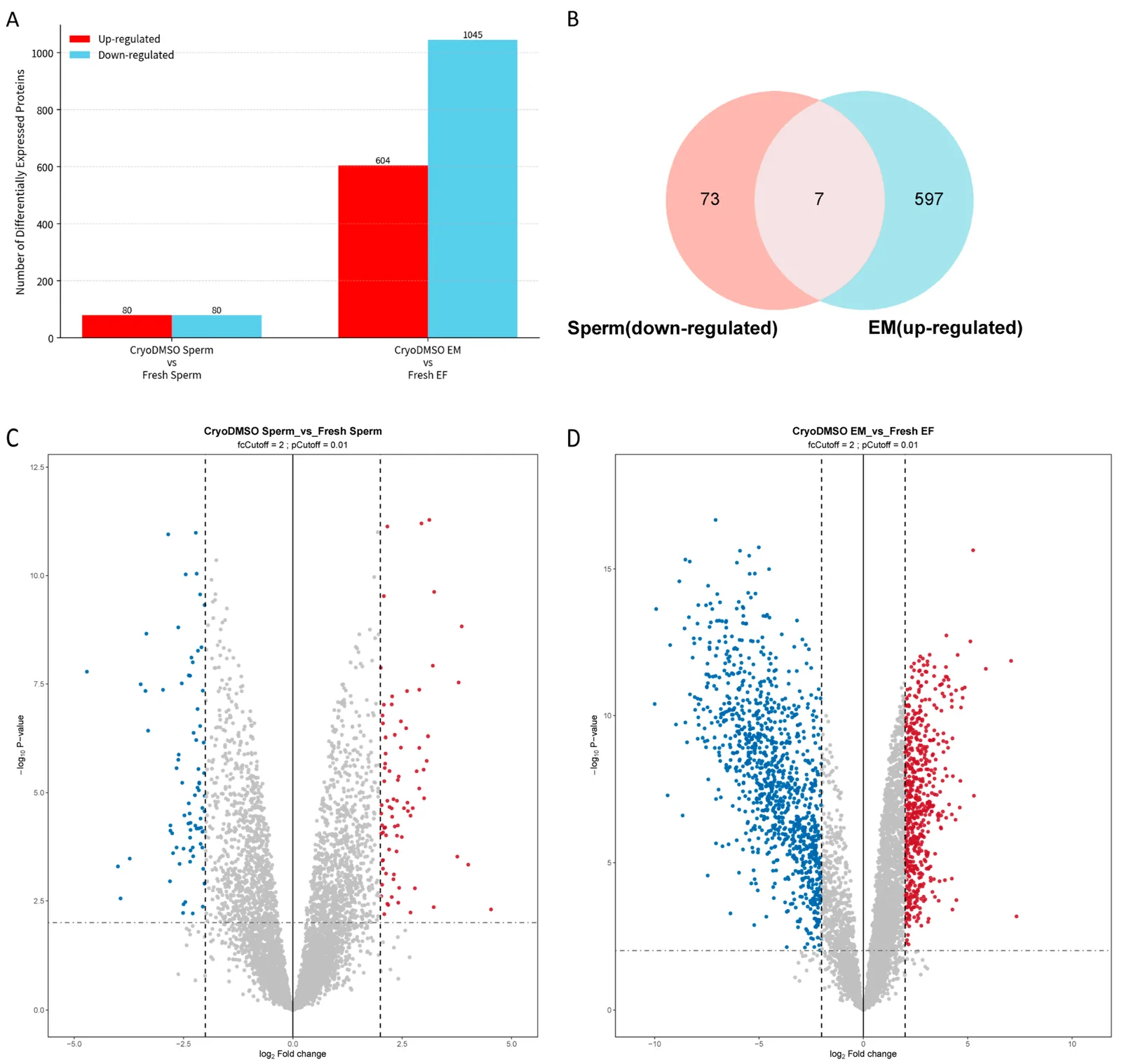
Analysis of leaked proteins in sperm before and after freezing. (**A**) Heatmap of DEPs in sperm samples before and after freezing; (**B**) Venn diagram of upregulated proteins in CryoDMSO EM and downregulated proteins in sperm after freezing; (**C**) Volcano plot of DEPs between fresh sperm and thawed sperm; (**D**) Volcano plot of DEPs between fresh EF and CryoDMSO EM. Blue dots: significantly downregulated proteins; Red dots: significantly upregulated proteins; Grey dots: proteins with no significant differential expression.

With a fold change (FC) > 2 and significance level *p* < 0.01 as screening criteria, the hierarchical clustering analysis results of differentially expressed proteins (DEPs) indicated close clustering relationships among replicate samples in each group, with good consistency of biological replicates (Figure 6A). Volcano plot analysis revealed a large number of significant DEPs between fresh sperm and cryopreserved sperm, as well as between fresh EF and CryoDMSO EM, and the number of upregulated proteins was significantly higher than that of downregulated proteins in both comparisons (Figure 6C,D). Through the identification of DEPs between fresh sperm and cryopreserved sperm, and between fresh EF and CryoDMSO EM, a total of 677 protein sets associated with sperm were screened (proteins downregulated in sperm and upregulated in CryoDMSO EM, Figure 6B). Compared with fresh sperm, 80 proteins were significantly downregulated in cryopreserved sperm, among which the most significantly downregulated proteins included PARP9, GMPPA, ARPC5, DRP2, and CROCC/Rootletin (Figure 6C). Compared with fresh EF, 604 proteins were significantly upregulated in CryoDMSO EM, among which the most significantly upregulated proteins included KATNAL2, DNM1L/DRP1, ACSF2, SMIM15, CNPY1, and Espin (Figure 6D). In addition, seven leaked proteins were simultaneously downregulated in sperm and upregulated in fresh EF (Figure 6B, Table 3), suggesting overlapping sperm leakage proteins induced by cryostress (Figure 6B).

### 2.6. GO and KGEE Enrichment Analysis of DEPs

**Figure 7:**
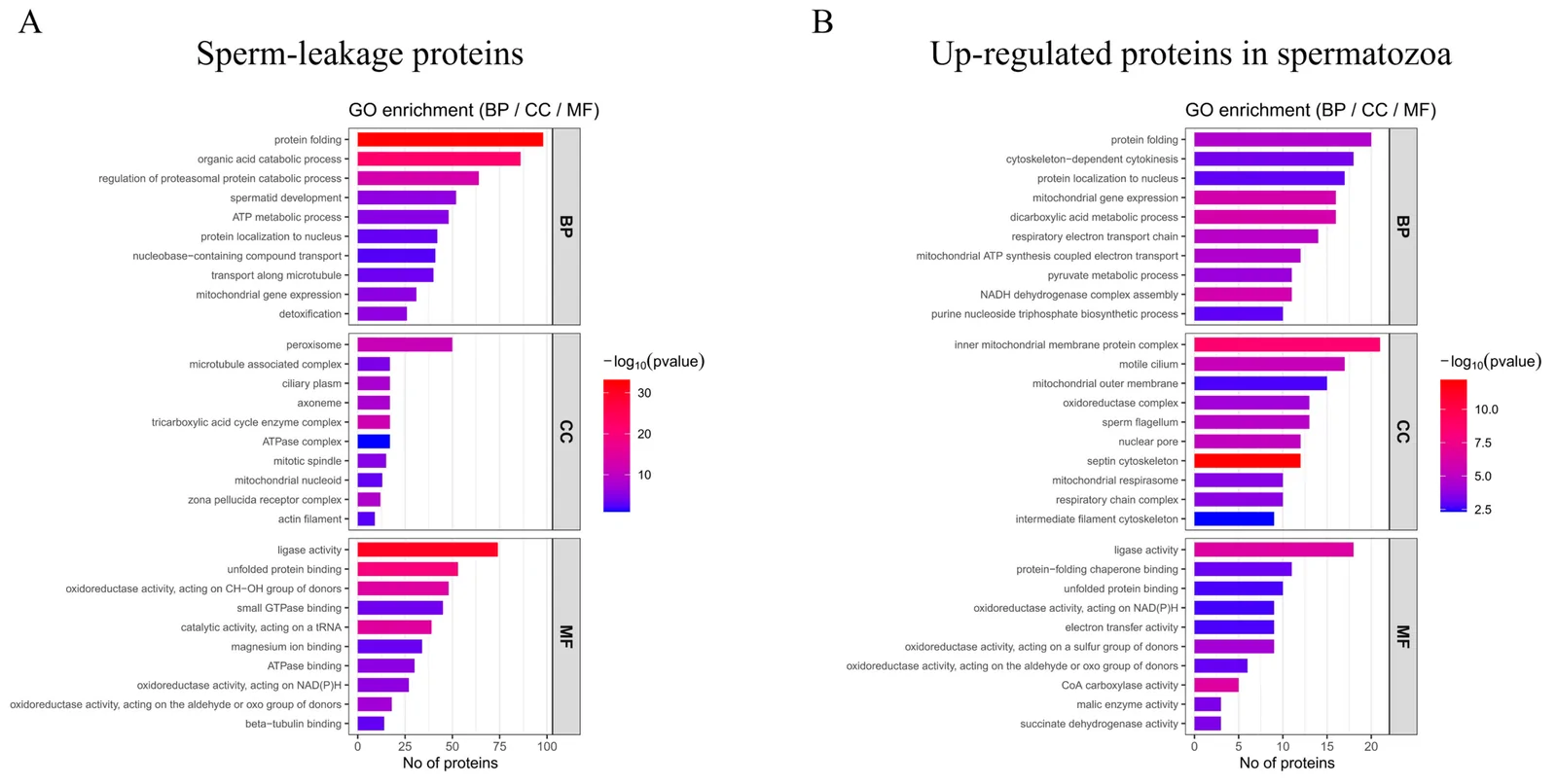
GO functional enrichment analysis of relatively enriched proteins within post-thaw spermatozoa from the D15 + 10% DMSO cryopreservation group. (**A**) GO enrichment analysis of leaked sperm proteins; (**B**) GO enrichment analysis of upregulated sperm proteins.

GO enrichment analysis was conducted on 677 proteins identified in sperm leakage (Figure 7A). Enrichment analysis revealed that biological processes relevant to sperm cryopreservation mainly comprised ATP metabolism, microtubule-mediated transport, and mitochondrial gene expression; molecular functions primarily involved catalytic and binding activities, including ATPase binding, β-tubulin binding, and NAD(P)H oxidoreductase activity; and cellular components included axonemes, ciliary matrix, microtubule-associated complexes, and actin filaments. Collectively, leaked proteins participate in energy metabolism and material transport, and largely originate from mitochondria and cilia, implying a close association with cryopreservation-induced structural damage in sperm. For significantly upregulated proteins in post-thaw sperm, GO enrichment revealed enriched biological processes, including protein folding, cytoskeleton-dependent cytokinesis, and nuclear protein localization (Figure 7B). Major molecular functions included ligase activity, chaperone and unfolded protein binding, NAD(P)H oxidoreductase activity, electron transfer activity, and coenzyme A carboxylase activity. Corresponding cellular components were enriched in mitochondrial inner membrane complexes, motile cilia, sperm flagella, oxidoreductase complexes, and respiratory chain complexes. These remodeled protein cohorts likely represent passive stress-responsive molecular signatures triggered by cryoinjury; they sustain sperm motility and energy supply via core physiological pathways to counteract cryodamage.

KEGG enrichment of DEPs identified prominent pathways, including endoplasmic reticulum (ER) protein processing, carbon metabolism, nucleotide metabolism, pyruvate metabolism, glycolysis/gluconeogenesis, the TCA cycle, 2-oxocarboxylic acid metabolism, and glutathione metabolism (Table 4). Carbon metabolism exhibited the highest enrichment significance (maximum −log_10_(P)) and the largest number of DEPs; both ER protein processing and carbon metabolism showed high gene ratios (~9%). Significant enrichment of energy metabolism pathways (glycolysis/gluconeogenesis, the TCA cycle) and oxidative stress-related glutathione metabolism indicated mild rearrangement of sperm intracellular energy metabolism and antioxidant systems upon cryostress. The major proteomic alteration induced by cryoinjury originated from massive protein efflux to the extracellular fraction.

## 3. Discussion

Sperm cryopreservation is an essential technique for germplasm resource conservation and artificial propagation of fish, yet ultra-low temperature freezing inevitably triggers complex physical and biochemical stresses, resulting in structural disruption, disturbed energy metabolism, and impaired molecular function in sperm [28]. In the present study, we systematically optimized diluent and cryoprotectant protocols for HFB sperm cryopreservation, comprehensively assessed post-thaw sperm quality in terms of macroscopic motility, cellular integrity, ultrastructural morphology, and proteomic profiles, and further explored key molecular pathways governing cryo-induced sperm damage and adaptive responses, providing a solid theoretical and technical foundation for standardized cryopreservation of HFB sperm.

Optimization of extenders and cryoprotectants is critical for successful sperm cryopreservation, yet the optimal combination of diluent and cryoprotectant is species-specific [14,17]. Among the extenders tested in this study, D15 exhibited the best preservation performance for HFB sperm, implying that it creates a suitable microenvironment to support sperm survival. By contrast, all sperm lost motility within 24 h in the Kurokura diluent, demonstrating its incompatibility with ultra-low temperature cryopreservation (ULTC). Studies have proven that low K^+^ concentrations block premature activation of sturgeon sperm, while high K^+^ levels drastically suppress sperm motility [29]. Furthermore, K^+^ in extenders imposes inhibitory effects on sperm movement in salmon and sturgeon species [29]. As a non-permeable saccharide, glucose not only regulates osmotic pressure and stabilizes membrane structure, but also supplies energy substrates to sustain basal sperm metabolism under cold storage, slowing motility loss [30,31,32]. Sperm can catabolize exogenous glucose to offset energy expenditure during locomotion, boost motility, and extend lifespan. Glucose supplementation experiments confirm that glucose improves sperm motility and alters its movement patterns [33]. Collectively, the Kurokura diluent contained excessive K^+^ that suppressed sperm motility; meanwhile, its lack of glucose disabled sustained cold-resistant metabolism, leading to energy exhaustion and complete sperm mortality. In comparison, D15 supplemented with an appropriate glucose concentration serves as a superior base diluent for HFB sperm cryopreservation.

DMSO possesses favorable membrane permeability and can rapidly penetrate sperm cells, bind water molecules to modulate intracellular osmotic pressure, and alleviate intracellular damage induced by ice crystal formation [34]. It has become the preferred cryoprotectant for sperm cryopreservation in most fish species, particularly cyprinids. Cryopreservation trials using DMSO on sperm of *Cyprinus carpio*, *Megalobrama amblycephala*, and other fish species have achieved post-thaw sperm motility of 60% [35]. Multiple investigations confirm that DMSO is applicable for sperm cryopreservation in diverse cyprinid fishes and yields optimal outcomes in terms of sperm motility and swimming velocity [35,36]. In the present study, 10% DMSO was identified as the most effective cryoprotectant among the four tested agents. Nevertheless, cryoprotectants confer protective effects on sperm while exhibiting cytotoxicity, which intensifies with rising concentration [37]. Consistent with this principle, the highest sperm motility was observed at 10% DMSO in this experiment, whereas motility declined markedly when DMSO concentration was elevated to 15% and 20%. Moreover, high DMSO cytotoxicity has been reported to severely impair sperm motility and fertilization capacity during cryopreservation of human sperm [38]. Therefore, an appropriate DMSO concentration is essential to balance cryoprotective efficacy and cytotoxicity for HFB sperm cryopreservation.

PMI, DI, MA, and ultrastructural characteristics serve as key indicators for evaluating sperm quality. In the present study, even with the optimized cryopreservation scheme, flow cytometry confirmed significant reductions in PMI, MA, and DI of post-thaw sperm, accompanied by evident ultrastructural impairments. The sperm plasma membrane not only safeguards cellular integrity but also mediates material exchange with the extracellular environment [39]. It is the main site where low-temperature damage occurs. Intracellular and extracellular ice crystal formation coupled with osmotic stress can directly rupture the lipid bilayer and trigger leakage of intracellular contents [40]. Sperm mitochondria produce substantial ATP through oxidative phosphorylation to power sperm motility and fertilization. Declining mitochondrial function further diminishes sperm motility, as corroborated by reduced VCL, VSL, and VAP values from CASA measurements. SEM and TEM observations demonstrated that HFB sperm share the typical teleost sperm architecture composed of a head, midpiece, and flagellum [41]. Following cryopreservation, multiple structural defects were detected, including disrupted plasma membranes, fractured flagella, swollen mitochondria with diminished or missing cristae, and decondensed chromatin. These morphological alterations closely matched the flow cytometric findings. Notably, despite such severe subcellular damage, sperm cryopreserved with the optimal combination of D15 extender and 10% DMSO exhibited fertilization and hatching rates statistically comparable to fresh sperm. This decoupling of structural integrity and fertilizing potential has been documented in other fish species. For *Gobiocypris rarus*, post-thaw sperm displayed markedly impaired PMI, MA, and DI relative to fresh sperm, yet achieved fertilization and hatching rates of 87.31% and 98.84%, respectively [42]. In *Gobiocypris rarus*, ultra-low temperature cryopreservation induced severe ultrastructural injuries [43]. However, fertilization and hatching rates did not differ significantly before and after cryopreservation. Collectively, these observations suggest that partially damaged sperm can still maintain fertilizing competence for oocytes [44]. Ultimately, successful fertilization only requires a limited number of sperm retaining intact fertilization capacity [45].

Alterations to the sperm genome mainly shape late embryonic development and larval survival, while the loss of sperm proteins impairs motility and fertilizing capacity, thereby disrupting early post-fertilization processes [46]. Mature sperm exhibit limited transcriptional activity, such that most biological processes rely on post-translational regulation at the protein level [47]. In the present study, 4D-DIA quantitative proteomics was applied to profile protein changes in fresh EF, CryoDMSO EM, fresh sperm, and CryoDMSO sperm, uncovering how cryopreservation reshapes the expression of sperm proteins. We identified 677 protein groups associated with cryostress-triggered protein leakage, including 80 significantly downregulated proteins in sperm and 604 upregulated proteins in the extracellular medium; seven proteins exhibited consistent leakage signatures. Key depleted proteins included PARP9, GMPPA, ARPC5, DRP2, and CROCC/Rootletin. Cryo-induced DNA lesions recruit PARP9-DTX3L complexes for DNA repair, accelerating PARP9 consumption, which corresponds to the decreased DI of post-thaw sperm [48,49,50]. CROCC/Rootletin is a coiled-coil protein present in the rootlets of ciliary cells [51]. Rootletin is one of the least dynamic structures among all cytoskeletons, interacts with actin filaments to provide structural support for cilia, and the stability of the centrosome connection in the sperm neck and the axoneme in the tail depends on Rootletin [52]. Osmotic fluctuations and ice crystal shear forces during cryopreservation disrupt the structural stability associated with Rootletin, indirectly aggravating sperm neck rupture, tail deformities, and other abnormalities, ultimately leading to decreased sperm quality. Concurrently, cryopreserved sperm activated compensatory responses via upregulated proteins represented by KATNAL2, DNM1L, ACSF2, SMIM15, CNPY1, and Espin. As a core regulator of microtubule cleavage and reorganization, KATNAL2 can accelerate the degradation and reassembly of damaged microtubules and functions in sperm head shaping, axoneme initiation, and ciliogenesis, maintaining normal sperm morphology and motility potential [53]. DNM1L/DRP1 governs mitochondrial fission and mitophagy to eliminate dysfunctional mitochondria, partially alleviating the loss of MA after thawing and sustaining energy supply for motility [54,55]. Espin promotes actin bundling to preserve ectoplasmic specialization junctions and sperm head morphology [56]. Collectively, these intracellularly retained and rearranged protein clusters constitute a passive molecular remodeling profile responding to cryoinjury, yet such compensation cannot fully offset structural and molecular damage, consistent with the compromised sperm quality detected by flow cytometry and ultrastructural observation. It should be noted that the increased abundance of 604 proteins in the CryoDMSO EM group may not be exclusively attributed to genuine protein leakage from spermatozoa. Differences in matrix composition introduced by cryoprotectant treatment, dilution effects during sample preparation, and alterations in the relative protein proportions between fresh and cryopreserved samples could all contribute to the observed signal shifts.

GO functional enrichment analysis of leakage-associated proteins illustrates the molecular characteristics corresponding to cryostress-induced structural damage in sperm. Leaked proteins were significantly enriched in energy metabolism, catalytic and binding activities such as ATP metabolism, organic acid catabolism, β-tubulin binding, axonemes, and ciliary matrices. This indicates that the targeted disruption and loss of leaked proteins involved in ATP metabolism and mitochondrial gene expression pathways directly compromise the integrity of the sperm energy supply system. As the core organelle of energy metabolism, mitochondria-related protein leakage may stem from cryopreservation-induced mitochondrial membrane rupture and structural disintegration, leading to insufficient ATP production and impaired energy supply for sperm motility and fertilization [57]. Proteins related to small GTPase and ATPase binding activities regulate cytoskeletal reorganization, material transport, and energy conversion [58,59]. Their loss may disrupt microtubule dynamic assembly and dynein function, resulting in reduced sperm motility. Leakage of proteins with β-tubulin binding activity is directly linked to the stability of the axonemal microtubule structure, which is highly consistent with ultrastructural damage such as flagellar fracture and axonemal disassembly observed after cryopreservation [60,61]. Leaked proteins associated with cytoskeletal structures such as axonemes, ciliary matrices, microtubule-associated complexes, and actin filaments are mostly key components maintaining sperm morphology and motility [56,62,63]. Their loss directly disrupts the integrity of the “9 + 2” microtubule structure, dynein arm activity, and actin cytoskeleton stability of the sperm flagellum, which is highly consistent with ultrastructural injuries such as sperm head rupture and flagellar fracture observed under SEM.

KEGG pathway enrichment analysis of differentially expressed proteins characterizes the global regulatory features of cryostress on sperm metabolic networks and cellular homeostasis. Carbon metabolism exhibited the highest enrichment significance and the greatest number of differentially expressed proteins. Together with the endoplasmic reticulum protein processing pathway, it achieved a gene ratio of nearly 9%, highlighting the central involvement of these two pathways in sperm responses to low-temperature stress. Serving as a core hub for substance metabolism and energy provision, carbon metabolism is broadly modulated, presumably as a compensatory strategy to counteract cryopreservation-triggered energy depletion [64]. Ice crystal injury and altered membrane permeability during freezing disturb energy metabolic equilibrium [65,66]. By coordinating downstream branches including glycolysis/gluconeogenesis, pyruvate metabolism, and the tricarboxylic acid cycle, carbon metabolism flexibly reshapes energy production [67]. Consistent with these metabolic regulatory characteristics, the extender containing an appropriate glucose concentration provides continuous energy substrates for sperm metabolic pathways under low-temperature stress, effectively stabilizing sperm energy metabolism and alleviating cryo-induced energy exhaustion.

Nevertheless, several limitations exist in the current research. The sample size was limited, and inter-male variation in cryotolerance was not assessed. A non-freezing cryoprotectant-only control was missing to separate reagent toxicity from freezing injury. In addition, partial extender screening depended on cold storage effects instead of post-thaw reproductive performance, candidate proteins from proteomics were not experimentally validated, post-hatching larval survival and quality data were not collected, and noticeable variability was found in post-thaw motility replicates. These shortcomings will be remedied in our subsequent expanded experiments to deepen the mechanistic study of fish sperm cryoinjury and protection.

## 4. Materials and Methods

### 4.1. Sample Collection

**Figure 8:**
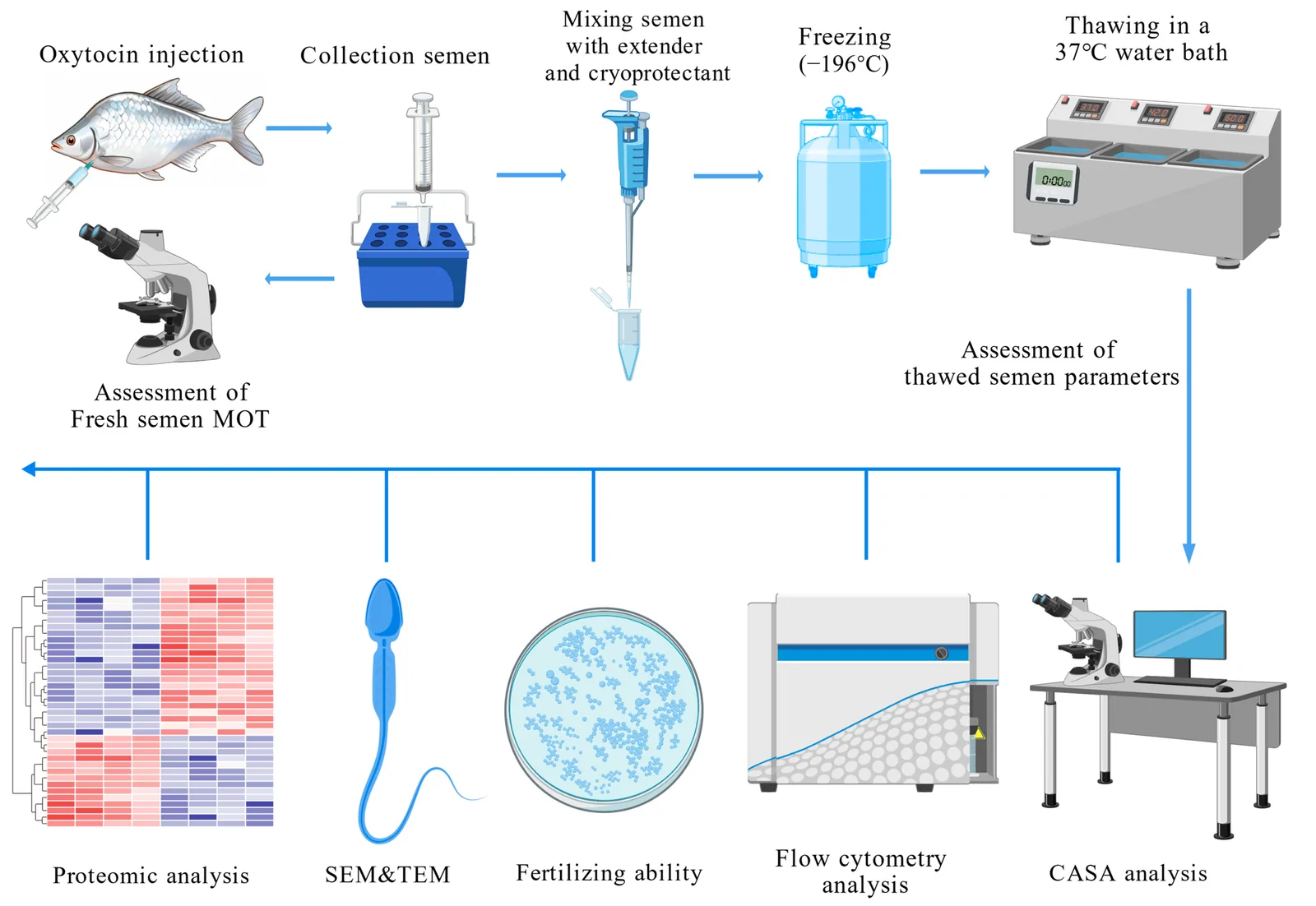
Experimental design for ultra-low temperature cryopreservation of HFB sperm.

The specimens were obtained from the Hunan Fish Genetics and Breeding Center of Hunan Normal University (Changsha, China). During the breeding season from April to June, five sexually mature and healthy individuals were carefully selected and then administered oxytocic agents to induce spawning. In the evening one day prior to semen collection, the selected male fish were transferred into the prepared aquaria. Human chorionic gonadotropin (HCG) at a dosage of 400 IU/kg body weight plus luteinizing hormone-releasing hormone A2 (LHRH-A2) at 7.50 μg/kg body weight was administered, with a latency period of approximately 24 h until stripping. Body surface water was wiped off with a dry and clean towel, and gentle abdominal pressure was applied to collect semen naturally. Semen was immediately transferred with a sterile syringe to centrifuge tubes pre-cooled to 4 °C in an ice bath, protected from direct light, and strictly free of blood, urine, or feces. Only samples with sperm motility > 90% under microscopic examination were used for ULTC experiments. All experimental procedures were approved by the Animal Care Committee of Hunan Normal University and followed the University’s guidelines for the welfare of experimental laboratory animals (GB/T 35823–2018) [68]. The experimental flow (Figure 8) is illustrated with BioGDP (https://biogdp.com/).

### 4.2. Determination of Sperm Density

Sperm density was counted using a hemocytometer. Fresh semen was diluted 1:1000 with PBS, and a drop of sperm suspension was loaded onto a hemocytometer (counting area: 16 × 25 grids, total volume: 0.1 mm^3^) covered with a coverslip. Sperm were counted under a microscope following the rule ‘count upper, not lower; left, not right’. Each sample was measured in triplicate. Sperm density (cells/mL) was calculated as: Sperm density = (Total sperm in 5 middle grids/5) × 25 × Dilution factor × 10^4^.

### 4.3. Screening of Extenders

Four extenders (D1, D15, D20, Kurokura) were prepared (Table 5) and pre-cooled at 4 °C. Semen was mixed with each diluent at a ratio of 1:5 and stored at 4 °C. At 5 min, 24 h, and 120 h, Cryopreserved sperm were activated by adding 3 μL of 0.65% NaCl activation solution to 2.5 μL of thawed semen on a glass slide, gently mixed, and covered with a coverslip. Sperm motility and kinematic characteristics were quantified using a CASA system (Hamilton Thorne Research, Beverly, MA, USA) equipped with an upright biological microscope and a 400× magnification objective lens. The minimum sperm head area was set at 5 μm^2^, and the maximum head area at 60 μm^2^; the acquisition rate was 60 frames per second, with continuous acquisition of 30 consecutive frames for trajectory reconstruction. Sperm activation rate is defined as the percentage of motile spermatozoa relative to the total sperm population after mixing the sperm sample with the activating solution. In the present study, the activation rate was adopted as the evaluation criterion for sperm motility. Sperm motility parameters were quantified within 15 s after activation, once the bulk flow of sperm ceased and approximately 100 spermatozoa were visible in the microscopic field. MOT, VSL, VCL, and VAP were recorded.

### 4.4. Screening of CPAs

Based on diluent screening, D15 was chosen as the base diluent. Four CPAs, methanol (MeOH), glycerol (Gly), dimethyl sulfoxide (DMSO), and ethylene glycol (EG), were prepared at volume fractions of 10%, 20%, 30%, and 40% with D15 and used after being freshly prepared. Semen was first mixed with D15 at 1:2, then mixed dropwise with CPA solutions at 1:1 to achieve final CPA concentrations of 5%, 10%, 15%, and 20%. After complete addition, the suspension was gently homogenized and equilibrated at 4 °C for 5 min to shorten sperm exposure to DMSO. The sperm–CPA suspensions were transferred into plastic straws using a syringe adapter, with a fixed loading volume of 0.25 mL per straw, sealed with plugs, and placed in a straw bag. Samples were equilibrated at 4 °C for 5 min, vapor-fumigated 10 cm above the liquid nitrogen surface for 10 min, floated on the liquid nitrogen surface for 5 min, then plunged directly into liquid nitrogen (–196 °C) for long-term storage. For thawing, straws were rapidly removed from liquid nitrogen, wrapped with plastic film, and fully immersed in a 37 °C water bath with gentle shaking for 13 s until ice crystals just melted. Thawed sperm were activated with 0.65% NaCl solution, and motility parameters were analyzed by CASA as described above.

### 4.5. Flow Cytometry Analysis

Sperm PMI, DI, and MA were assessed using a BD LSRFortessa flow cytometer (BD Biosciences, Franklin Lakes, NJ, USA). The instrument was configured with a 488 nm blue laser and a 633 nm red laser, paired with the following bandpass filters: 530/30 nm for SYBR-14 and Rh123, 610/20 nm for propidium iodide (PI), and 575/25 nm for acridine orange (AO). PMI, DI, and MA were evaluated via flow cytometry with different staining protocols. Briefly, sperm samples were uniformly diluted to a concentration of 3 × 10^7^ cells/mL using PBS for subsequent staining and detection. For PMI assessment, sperm were stained with 100 nmol/L SYBR-14, thoroughly mixed, and incubated in the dark at room temperature for 10 min, followed by the addition of 12 μmol/L PI and another 10 min of dark incubation. In SYBR-14/PI plots, SYBR-14^+^PI^+^ and SYBR-14^−^ PI^+^ were categorized as membrane-damaged sperm, and only SYBR-14^+^ PI^−^ events were counted as intact sperm for PMI calculation. Subjective descriptors such as ‘cells in a state of impending death’ were eliminated entirely. For DI detection, prior to AO staining, sperm pellets were resuspended in acid detergent denaturation solution and incubated for 30 s to induce unwinding of broken DNA strands according to standard SCSA procedures. Sperm were single-stained with 100 μg/mL AO and incubated under the same dark and room temperature conditions for 10 min. For MA measurement, sperm were co-stained with 10 μg/mL Rh123 and 12 μmol/L PI, with two consecutive 10 min dark incubation steps consistent with the PMI staining procedure. In Rh123/PI plots, Rh123^+^ PI^−^ events represented viable sperm with intact mitochondrial membrane potential, while Rh123^−^PI^+^ indicated non-viable sperm with lost mitochondrial function, with no indirect physiological deduction added. After staining, all samples were washed twice with PBS, centrifuged at 1000× *g* for 5 min at 4 °C, and resuspended in PBS. Finally, the fluorescence intensity of the corresponding dyes was detected and analyzed by flow cytometry to determine sperm PMI, DI, and MA, respectively. Flow cytometric raw data were acquired using BD FACSDiva (BD Biosciences, NJ, USA). First, sperm-related events were distinguished from background debris via forward-scatter versus side-scatter. The single-cell sperm population was further screened to eliminate diploid cells and cell aggregates. Within singlet sperm populations, fluorescence gates were determined with the aid of unstained negative controls and single-color compensation controls. Subpopulation gates were manually drawn according to the fluorescence distribution profiles of sperm events in dot plots.

### 4.6. Fertilization

Mature female blunt snout bream and male HFB were induced to spawn the night before insemination. Eggs were collected by gentle abdominal pressure. A unified sperm-to-egg ratio of 1 × 10^7^ spermatozoa per egg was applied to each fertilization group to ensure equal sperm dosage for valid comparison. Fresh semen and thawed semen preserved with D15 and 10% DMSO were separately mixed with eggs to achieve fertilization. For the fresh sperm group, eggs were activated with a small amount of aerated water after adding undiluted raw semen and were fully blended with a feather. For the cryopreserved group, volume compensation was implemented to counteract the 1:5 dilution factor during sperm cryopreservation. Fertilized eggs were incubated in Petri dishes at room temperature. Dead eggs were removed, and the water was replaced every three hours. Fertilization rate was calculated at the mid-gastrula stage; hatching rate was calculated after hatching. Each group was tested in triplicate. Fertilization rate (%) = (Number of gastrula embryos/Total number of eggs) × 100%. Hatching rate (%) = (Number of hatched larvae/Total number of fertilized eggs) × 100%.

### 4.7. Ultrastructural Observation

For SEM and TEM analysis, fresh sperm and post-thaw sperm cryopreserved in D15 diluent supplemented with 10% DMSO were first fixed in 2.5% glutaraldehyde (1:10 dilution) at room temperature in the dark for 2 h, followed by temporary storage at 4 °C. All fixed samples were rinsed with PBS (pH 7.4), post-fixed in 1% osmic acid at 4 °C in the dark for 2 h, and washed again prior to dehydration with a graded ethanol series. For SEM observation, dehydrated samples were dried with a CO_2_ critical point dryer, sputter-coated with gold using an ion coater, and subsequently imaged under an SEM. For TEM analysis, dehydrated specimens were infiltrated and embedded in epoxy resin; ultrathin sections were prepared, double-stained with uranyl acetate and lead citrate, and finally examined and photographed with a TEM.

### 4.8. Proteomic Analysis

4D-DIA quantitative proteomics was used to identify differential proteins between fresh and cryopreserved sperm. Fresh semen and semen cryopreserved with D15 and 10% DMSO were centrifuged at 1000× *g* for 10 min at 4 °C twice to obtain total protein solutions of fresh extracellular medium (fresh EF) and frozen extracellular medium (CryoDMSO EM). Sperm pellets were washed with PBS, fully ground in liquid nitrogen, and lysed in lysis buffer containing phosphatase and protease inhibitors. After ultrasonication on ice (80 W, 1 s on/1 s off for 2 min) and centrifugation, supernatants containing total sperm protein were collected, which were designated as total protein solutions of fresh sperm and frozen sperm (CryoDMSO). After the protein was digested by trypsin and subjected to peptide salting-out, it was then analyzed by mass spectrometry and bioinformatics. Total protein concentration was determined using the BCA protein quantification kit, following the manufacturer’s instructions after thawing the stored frozen protein samples. A total of 50 μg of quantified protein was subjected to subsequent denaturation, magnetic bead purification, and tryptic digestion. The protein concentrations for analysis were as follows: fresh sperm (4453 ± 83) μg/mL, frozen sperm (3862 ± 61) μg/mL, Fresh EF (742 ± 17) μg/mL, CryoDMSO EM (806.15 ± 27) μg/mL. Vacuum-dried peptides were redissolved in Nano-HPLC Buffer A and separated on an EASY 1200 nano-UHPLC (Thermo, Waltham, MA, USA) with trap and analytical C18 columns. Mobile phases contained 0.1% formic acid in water (A) and 0.1%/80% acetonitrile (B). Peptides were enriched and eluted at 600 nL/min via a 24 min linear gradient (8–35% B over 0–21 min, ramped to 100% B and held). Eluates were analyzed on a timsTOF HT mass spectrometer (Bruker Daltonics, Bremen, Germany) in DIA mode with variable *m*/*z* 300–1400 isolation windows for 4D measurement. Owing to the lack of a dedicated database for the hybrid fish, the UniProt *Cyprinus carpio* reference library was used. Raw spectra were processed with DIA-NN v1.8.1: trypsin digestion (max 1 missed cleavage), fixed cysteine carbamidomethylation, variable N-terminal acetylation and methionine oxidation, with auto mass tolerance calibration. Differentially expressed proteins (DEPs) were screened based on the criteria of *p*-value < 0.01 and |Log2FC| > 2. Functional annotation and pathway enrichment analyses of DEPs were performed using Gene Ontology (GO) and Kyoto Encyclopedia of the Genes and Genomes (KEGG) databases, respectively.

### 4.9. Statistical Analysis

All quantitative data were statistically analyzed using SPSS 27.0 software. Prior to comparative analysis, the Shapiro–Wilk test was used to assess the normal distribution of datasets. One-way ANOVA was used to evaluate intergroup differences. Homogeneity of variances was assessed by Levene’s test; where the assumption held (*p* > 0.05), Tukey’s HSD was applied for post hoc pairwise comparisons. Unless otherwise stated, three independent male HFB were used as biological replicates for each treatment (*n* = 3), and one technical replicate was analyzed for the sperm sample derived from each individual fish. Differences with *p* < 0.05 were regarded as statistically significant. All measurement data were presented as mean ± SD. Bar charts were plotted using the ggplot2 package in R with self-edited customized scripts.

## 5. Conclusions

In this study, we optimized the low-temperature and cryopreservation protocols for HFB sperm by evaluating sperm motility and kinematic parameters, and screened D15 extender and 10% DMSO as the optimal preservation system. We further comprehensively assessed post-thaw sperm quality via flow cytometry, SEM, and TEM to detect cellular activity and ultrastructural changes. Additionally, 4D-DIA quantitative proteomics was applied to identify cryostress-induced differentially expressed proteins and related functional pathways. Collectively, this study systematically clarifies the physiological, structural, and molecular damage to HFB sperm caused by cryopreservation. It establishes an efficient cryopreservation technique for HFB sperm and provides novel mechanistic insights into fish sperm cryodamage, supporting germplasm resource conservation and artificial breeding of HFB.

## Figures and Tables

**Table 1:** Comparison of HFB sperm motility in different extender.

Extender	Sperm Motility (MOT, %)		
	5 min	24 h	120 h
Fresh	98.87 ± 1.40 ^a^	-	-
D1	95.40 ± 1.92 ^a^	97.10 ± 2.15 ^a^	-
D15	95.43 ± 3.36 ^a^	98.37 ± 1.48 ^a^	14.92 ± 2.25
D20	96.47 ± 3.78 ^a^	91.13 ± 3.17 ^b^	-
Kurokura	77.80 ± 7.60 ^b^	-	-

Note: Values in the same column with different lowercase superscript letters indicate significant differences (*p* < 0.05); “-” indicates the value is zero. Each treatment included three independent biological replicates (*n* = 3).

**Table 2:** Sperm motility parameters during 4 °C storage in different extender.

Extender		Kinematic Parameters (4 °C, μm/s)		
		5 min	24 h	120 h
Fresh	VCL	118.87 ± 5.38 ^a^	-	-
	VSL	86.18 ± 5.67 ^a^	-	-
	VAP	107.48 ± 4.23 ^a^	-	-
D1	VCL	107.60 ± 13.14 ^a^	113.89 ± 9.3 ^a^	-
	VSL	85.79 ± 7.85 ^a^	82.69 ± 5.63 ^a^	-
	VAP	99.37 ± 11.57 ^a^	101.94 ± 12.61 ^a^	-
D15	VCL	111.95 ± 18.25 ^a^	107.04 ± 9.5 ^a^	41.06 ± 4.45
	VSL	85.02 ± 2.71 ^a^	81.21 ± 6.11 ^a^	28.61 ± 2.92
	VAP	103.30 ± 14.02 ^a^	98.01 ± 13.31 ^a^	38.84 ± 4.69
D20	VCL	101.24 ± 12.10 ^a^	83.87 ± 9.52 ^b^	-
	VSL	66.74 ± 12.25 ^b^	62.42 ± 5.81 ^b^	-
	VAP	89.50 ± 18.83 ^a^	76.46 ± 9.40 ^b^	-
Kurokura	VCL	37.61 ± 5.37 ^b^	-	-
	VSL	29.23 ± 4.51 ^c^	-	-
	VAP	33.08 ± 4.95 ^b^	-	-

Note: In the same column, values with different lowercase letters superscripts mean significant difference (*p* < 0.05); “-” indicates that this data is 0. Each treatment was conducted with three independent biological replicates (*n* = 3).

**Table 3:** The seven core leakage proteins identified by proteomic comparison.

Protein	Sperm_log_2_FC	Sperm_*p*-Value	Medium_log_2_FC	Medium_ *p*-Value
PSMD5	2.20	6.76 × 10^−5^	−2.08	1.15 × 10^−4^
TIP20	2.00	7.96 × 10^−5^	−2.20	2.99 × 10^−5^
C9orf85	2.04	7.02 × 10^−7^	−2.40	9.60 × 10^−8^
CROCC	2.80	7.56 × 10^−5^	−2.48	2.43 × 10^−4^
ABI2B	2.66	2.73 × 10^−6^	−2.84	1.22 × 10^−6^
GMPPA	3.48	3.20 × 10^−8^	−2.97	2.38 × 10^−7^
SMIM15	4.00	5.06 × 10^−4^	−4.46	1.89 × 10^−4^

**Table 4:** Top 10 pathways of leaked sperm proteins by KEGG analysis.

Pathway	*p*-Value	Number of Proteins
Carbon metabolism	2.92 × 10^−25^	76
Protein processing in endoplasmic reticulum	4.74 × 10^−10^	76
Nucleotide metabolism	5.17 × 10^−10^	46
Pyruvate metabolism	8.57 × 10^−11^	30
Glycolysis/Gluconeogenesis	6.27 × 10^−7^	30
Citrate cycle (TCA cycle)	4.92 × 10^−15^	29
2-Oxocarboxylic acid metabolism	1.31 × 10^−12^	28
Glutathione metabolism	5.42 × 10^−3^	18
Pentose phosphate pathway	3.09 × 10^−5^	16
Nucleocytoplasmic transport	9.89 × 10^−4^	14

**Table 5:** Composition of diluent for ultra-low temperature cryopreservation of HFB sperm.

Component (g/L)	Extender			
	D1	D15	D20	Kurokura [69]
NaCl	7.8	8	8	3.6
KCl	0.5	0.5	1	10
CaCl_2_	-	-	-	0.22
NaHCO_3_	-	-	-	0.2
MgCl_2_	-	-	-	0.08
Glucose	15	15	15	-

Note: “-”: The corresponding substance was not added.

## Data Availability

The original contributions presented in the study are included in the article. Further inquiries can be directed to the corresponding author (yangch@hunnu.edu.cn). Differential protein lists and supporting proteomics data are publicly available via Mendeley Data (https://doi.org/10.17632/9tdnr2kd33.1).

## Abbreviations

The following abbreviations are used in this manuscript:

HFB	Hefang bream
DMSO	Dimethyl sulfoxide
SEM	Scanning electron microscopy
TEM	Transmission electron microscopy
EG	Ethylene glycol
CASA	Computer-assisted sperm analysis
MOT	Motility
VAP	Average path velocity
PMI	Plasma membrane integrity
DI	DNA integrity
MA	Mitochondrial activity
VCL	Curvilinear velocity
VSL	Straight-line velocity
fresh EF	Fresh extracellular medium
CryoDMSO EM	Frozen extracellular medium
CryoDMSO	Frozen sperm
AO	Acridine orange
Rh123	Rhodamine 123
